# *CYP2C19* Polymorphism in Ischemic Heart Disease Patients Taking Clopidogrel After Percutaneous Coronary Intervention in Egypt

**DOI:** 10.1007/s44197-023-00113-4

**Published:** 2023-05-18

**Authors:** Ahmed Shawky, Hussein Sabit, Mahmoud Nazih, Khalid Baraka, Mokhtar El-Zawahry

**Affiliations:** 1 Department of Cardiology, College of Medicine, Helwan University, Cairo, Egypt; 2 Department of Medical Biotechnology, College of Biotechnology, Misr University for Science and Technology, P. O. Box 77, Giza, Egypt; 3 Department of Clinical Pharmacy, Faculty of Pharmacy, Menoufia University, Shibin Al Kawm, Egypt; 4 Scientific Office, Egyptian Society of Pharmacogenomics and Personalized Medicine (ESPM), Cairo, Egypt; 5 Department of Cardiology, College of Medicine, Minia University, Minia, Egypt

**Keywords:** Cardiovascular, PCI, Clopidogrel, Egyptian, CYP2C19, Genotyping

## Abstract

**Background:**

Cardiovascular diseases (CVDs) are considered a leading cause of death worldwide. Allelic variation in the *CYP2C19* gene leads to a dysfunctional enzyme, and patients with this loss-of-function allele will have an impaired clopidogrel metabolism, which eventually results in major adverse cardiovascular events (MACE). Ischemic heart disease patients (*n* = 102) who underwent percutaneous cardiac intervention (PCI) followed by clopidogrel were enrolled in the present study.

**Methods:**

The genetic variations in the *CYP2C19* gene were identified using the TaqMan chemistry-based qPCR technique. Patients were followed up for 1 year to monitor MACE, and the correlations between the allelic variations in *CYP2C19* and MACE were recorded.

**Results:**

During the follow-up, we reported 64 patients without MACE (29 with unstable angina (UA), 8 with myocadiac infarction (MI), 1 patient with non-STEMI, and 1 patient with ischemic dilated cardiomyopathy (IDC)). Genotyping of *CYP2C19* in the patients who underwent PCI and were treated with clopidogrel revealed that 50 patients (49%) were normal metabolizers for clopidogrel with genotype CYP2C19*1/*1 and 52 patients (51%) were abnormal metabolizers, with genotypes CYP2C19*1/*2 (*n* = 15), CYP2C19*1/*3 (*n* = 1), CYP2C19*1/*17 (*n* = 35), and CYP2C19*2/*17 (*n* = 1). Demographic data indicated that age and residency were significantly associated with abnormal clopidogrel metabolism. Moreover, diabetes, hypertension, and cigarette smoking were significantly associated with the abnormal metabolism of clopidogrel. These data shed light on the inter-ethnic variation in metabolizing clopidogrel based on the *CYP2C19* allelic distribution.

**Conclusion:**

This study, along with other studies that address genotype variation of clopidogrel-metabolizing enzymes, might pave the way for further understanding of the pharmacogenetic background of CVD-related drugs.

## Introduction

Cardiovascular disease (CVD) is a class of diseases that affect the heart or blood vessels. Heart failure (HF) is a prevalent clinical condition representing various heart diseases [1, 2]. CVDs are the leading cause of death globally, with an estimated 17.9 million people dying from CVDs in 2019, accounting for 32% of all global deaths. It is expected to cause death for nearly 23.6 million in 2030 [3]. Among the spectrum of CVDs, coronary artery disease (CAD) is caused by atherosclerosis. This condition restricts blood flow to the heart, resulting in a heart attack when the blood flow is completely hindered. Atherosclerosis occurs upon a cascade of events, including vascular injury, inflammation, degeneration, and thrombosis [4, 5].

For the management of atherosclerosis, percutaneous coronary intervention (PCI) is the method of choice. This intervention requires antiplatelet therapy depending on the admission diagnosis and the stent type [6, 7]. Antiplatelet drugs are commonly used to control ischemic events, mainly in patients with acute coronary syndromes (ACS) [8, 9]. Despite the ongoing development of new antiplatelet drugs, variability in response has been observed, leading to adverse cardiovascular events [10, 11].

The interindividual pharmacogenetic variation in response to approved antiplatelet drugs has been extensively studied, emphasizing the antiplatelet prodrug clopidogrel. These drugs are for asymptomatic peripheral arterial disease (PAD), ischemic stroke, and ischemic events caused by ACS. Several factors can affect the platelet activation process, including epinephrine, thrombin, serotonin, and collagen. These factors have been extensively studied as they represent the site of action for antiplatelet drugs [11, 12].

Clopidogrel is a commonly used antiplatelet prodrug, converted in the liver into its active form that functions by targeting platelet P2Y12 receptor and inhibiting ADP-mediated platelet activation and aggregation [13, 14]. About 85% of the clopidogrel prodrug is hydrolyzed to inactive metabolites by esterase, while only 15% is transformed into active drugs. Two sequential oxidative reactions are necessary for this transformation [15, 16]. Clopidogrel combined with aspirin reduces cardiovascular-related death and ischemic complications in patients with ACS. However, interindividual variations in platelet aggregation are expected, leading to thrombotic events in some patients [17, 18].

Cytochromes P450 (*CYP*) are a superfamily of monooxygenases that, in humans, oxidize steroids, fatty acids, and xenobiotics and are essential for detoxification and clearance of a variety of drugs with toxicological importance [17, 18]. The human genome contains 57 genes and 59 pseudogenes that comprise 18 *CYP* families and 43 subfamilies. Several members in this family, such as *CYP1, CYP2, and CYP3* of *CYP450*, received more attention because of their significant role in pharmacokinetics [19]. In humans, 4 *CYP2C* members have been identified; *CYP2C8*, *CYP2C9*, *CYP2C18*, and *CYP2C19*. *CYP2C19* is a hepatic cytochrome P450 that metabolizes various clinical drugs, including clopidogrel, with 35 alleles. The CYP2C19*1 allele is the wild-type copy with full enzymatic activity. Other alleles include CYP2C19*2, which encodes for a truncated, nonfunctional protein, and CYP2C19*3, which harbors a premature stop codon. These polymorphisms of *CYP2C19* are associated with the abnormal metabolism of clopidogrel [20].

Ethnicity can influence the risk of phenoconversion, a phenomenon that might affect genotype-based drug prescription [21], where ethnicity can determine drug response and metabolism [22].

In the present study, we evaluated the role of *CYP2C19* allelic variation in the metabolism of clopidogrel in an Egyptian cohort population with CAD.

## Patients and methods

### Study population

In this descriptive cross-sectional study, 102 patients attended ELDOA Hospital, the Cardiovascular Department of Beni Suef University Hospital, and Cairo Health Care Center for Cardiac Catheterization for Percutaneous Coronary Intervention (PCI) from April 2017 to April 2018 were enrolled. Samples were represented as the main Governorates in Egypt. The study was approved by the IRB, Misr University for Science and Technology. Formally informed consent was collected from each participant after explaining the aim and objectives of the study.

In this study, patients with ACS who underwent PCI followed by clopidogrel treatment met the inclusion criteria. Patients on aspirin, clopidogrel, contrast allergy, Bare-Metal Stents (BMS) stents due to a higher risk of thrombosis, tumors or bleeding tendency, hemodynamic instability, and severe renal or liver dysfunction were excluded from this study.

Data were recorded blood sample was collected after obtaining informed consent from the patient. Personal data were verbally collected from all patients (name, age, address, education level, patient history, and family history of heart disease). Current and previous and follow-up clinical and laboratory data were collected from the medical record of the patients, which includes ECG reports, echocardiographic reports, and angiographic reports. Similarly, the collected data included the medications and doses administered, including clopidogrel and the reason for clopidogrel intake.

### Clinical and laboratory investigations

Family and medical history were collected from each patient. According to the American Heart Association guidelines, patients with blood pressure higher than 130/80 mm Hg were considered hypertensive patients. Furthermore, fasting and 2 h postprandial blood glucose levels were recorded for each patient. Patients with fasting glucose levels of 126 mg/dL and 2 h postprandial (two h pp) blood glucose levels above or equal to 220 mg/dL were considered people with diabetes according to the American Diabetes Association guidelines. Troponin (for the diagnosis of STEMI and non-STEMI), CK-MB, and CBC were also measured. Moreover, imaging investigations including ECG, Echocardiography, and Coronary angiography were performed for each patient (data not shown).

### PCI and clopidogrel management

The standard of care performed PCI. All patients were administrated 300 mg clopidogrel and 40 mg atorvastatin before operation and operated according to the type of anticoagulant, the use of glycoprotein IIb/IIIa inhibitor (tirofiban), and the type of stent. After PCI, patients were treated with 100 mg/day of aspirin and 75 mg/day of clopidogrel for 1 year. The success of PCI was defined as follows: (1) achievement of complete revascularization and residual stenosis of < 20% in target vessels of patients, (2) level 3 TIMI blood flow, (3) partial or complete remission of symptoms of myocardial ischemia, and (4) no severe complications during hospitalization (such as acute myocardial infarction, urgent target lesion revascularization, or death).

### Chemicals and kits

Taq-AT Polymerase, PCR buffer, mineral oil, the polymorphisms under study [*CYP2C19*:681G>A*2 (P227P)-960MCL, *CYP2C19*:636 G>A*3 (W212X)-960MCL, *CYP2C19*:806 C>T *17-960MCL], and DNA extraction kits were purchased from DNA Technology, Russia. The identification of the studied polymorphisms was performed at the laboratory of the Biotechnology Department, GMA Biotechnology, Cairo, Egypt.

### Molecular analysis

Approximately 2.5 mL of venous blood samples on heparin-coated tubes from 102 patients. Total DNA was extracted using a DNA extraction kit (prep-rapid Genetics, DNA Technology, Russia). For the qPCR amplification, the following mix was prepared for each SNP [*CYP2C19* 681 G>A (*2), *CYP2C19* 636 G>A (*3), *CYP2C19* -806 (*17)] in a total volume of 50 μL: 20 μL from each specific probe, 10 μL master mix, 15 μL mineral oil, and 5 μL DNA (equals to 500 ng).

### Interpretation of CYP2C19 alleles

Each of the two variant alleles is caused by a substitution of one nucleotide leading to different effects in the enzymatic activity where CYP2C19*1 is the wild type, CYP2C19*2 characterized by c.681G>A (rs4244285), which leads to cryptic splice acceptor activation, and CYP2C19*3 characterized by c.636G>A (rs4986893) which leads to gained stop codon. The predicted metabolism phenotypes for *CYP2C19* were: extensive metabolizer (CYP2C9-1*/1*), intermediate metabolizer (CYP2C9-1*/2*, CYP2C9-1*/3*, and poor metabolizer (CYP2C9-2*/2*, CYP2C9-2*/3*, and CYP2C9-3*/3*).

### Statistical analysis

Data were entered in a spreadsheet using (SPSS v.22, Chicago, Illinois, USA) program. Discrete values were presented in numbers, and the chi-square test made percentages and inferences of significance and the Fisher exact test. Quantitative data were expressed as mean ± standard deviation (SD). Qualitative data were expressed as frequency and percentage. Continuous variables were presented in means ± , standard deviation, the Student *t* test for normally distributed data, and the Kruskal–Wallis test for non-normal distribution data. The significance level was set at *p* < 0.05. All data analysis was done by the statistical package for social sciences (SPSS v.22, Chicago, Illinois, USA).

## Results

### Demographic data

In the present study, 102 patients were enrolled, and their demographic data are represented in Table 1.

**Table 1:** Demographic data distribution of the study group

Demographic data (*n* = 102)					
Sex		Age (years)		Residency*	
Males	Females	< 60 years	≥ 60 years	Rural	Urban
64 (62.75%)	38 (37.25%)	57 (55.88%)	45 (44.12%)	46 (45.1%)	56 (45.9%)

*Categorized as urban or rural based on the World Health Organization's definition of urban and rural areas

**Fig. 1:**
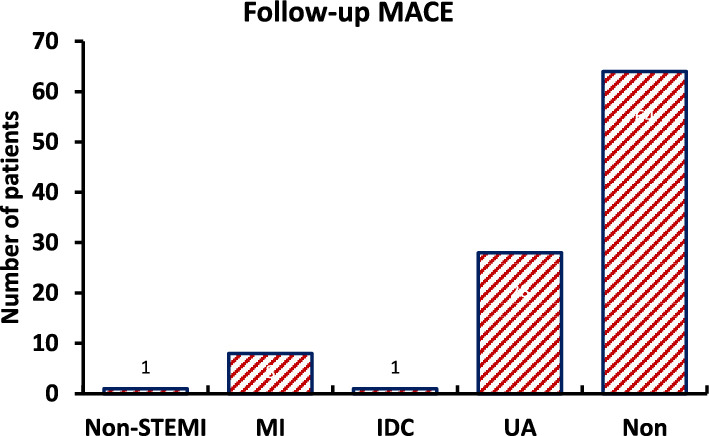
Follow-up MACE distribution of the study group. *MI* Myocardial Infarction, *IDC* Ischemic Dilated Cardiomyopathy, *UA* Unstable Angina

For the risk factors in the study group, 49 (48.04%) were found to have diabetes mellites type 2, 56 (54.9%) were having hypertension, and 40 (39.22%) were smokers. Figure 1 represents the study group’s major adverse cardiovascular events (MACE) during 1 year of follow-up.

The *CYP2C19* allelic distribution was variably distributed in the study group, with the most common genotype *1/*1, which represented 49 (48.04%) of the total number of patients followed by *1/*17 which represented 35 (34.31%) of the total number of patients.

According to the variation in genotypes, phenotype distributions were also recorded. Table 2 represents the distribution of the real-life experience of the patients enrolled in this study in terms of the capability of metabolizing clopidogrel. Data showed that 50 patients (49.25%) were normal metabolizers of clopidogrel, while 52 patients representing 50.98% of the study group were abnormal metabolizers. Table 3 represents the allelic and genotype frequency of the study population.

**Table 2:** Genotype distribution and phenotypes of the study group

Phenotype/genotype	Total (*n* = 102)
Abnormal metabolizer (*1/*2, *1/*3, *1/*17, *2/*17)	52 (50.98%)
Normal metabolizer (*1/*1)	50 (49.02%)

**Table 3:** The allelic and genotype frequency in the study population

Polymorphism	Allelic frequency	Genotype frequency
CYP2C19*2	15.3%	N/A
CYP2C19*3	1.2%	N/A
CYP2C19*17	30.39%	N/A

**Fig. 2:**
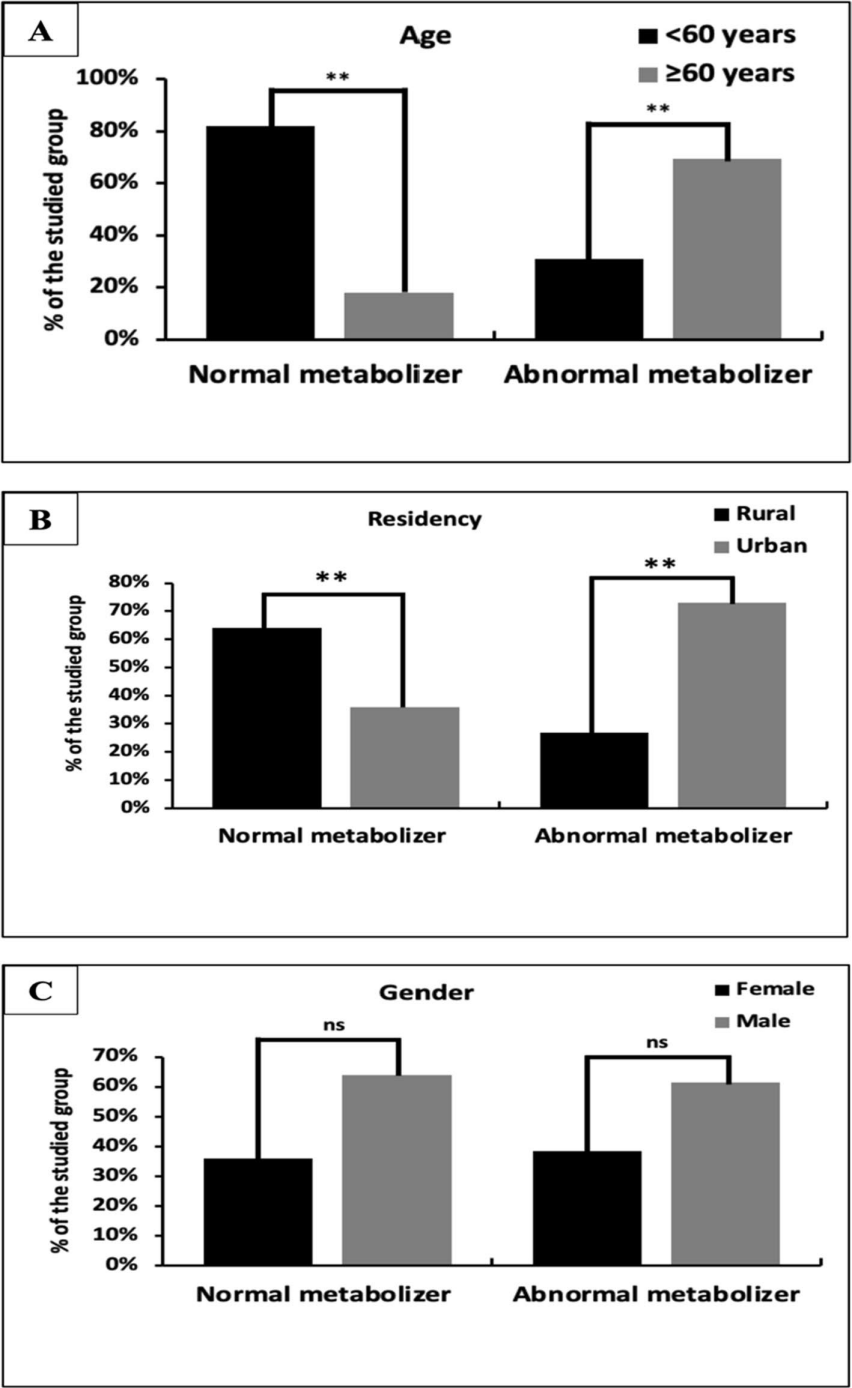
Comparison of normal and abnormal metabolizers according to demographic data

We linked the normal and abnormal metabolizers with their demographic data to gain a deeper insight. The obtained data (Fig. 2) indicated that there are significant variations for age (*p* < 0.001) (Fig. 2A) and residency (*p* < 0.001) (Fig. 2B), while sex did not yield significant differences (*p* = 0.797) (Fig. 2C). Age was a determinant factor in terms of the capability to metabolize clopidogrel.

Patients over 60 years old at the time of testing (*n* = 45) were 9 (18%) normal metabolizers and 36 (69.2%) abnormal metabolizers. While patients below 60 years old (*n* = 57) were 41 (82%) normal metabolizers and 16 (30.8%) abnormal metabolizers. Meanwhile, the residency also played a role in determining the patient’s capability to metabolize clopidogrel. Data indicated that out of 50 patients who normally metabolize the drug, 32 patients (61%) lived in a rural area, while 18 patients (36%) lived in an urban area. On the other hand, out of the 52 patients who abnormally metabolize clopidogrel, 14 (26.9%) were from rural areas, and the rest 38 patients (73.1%) were from urban areas.

**Fig. 3:**
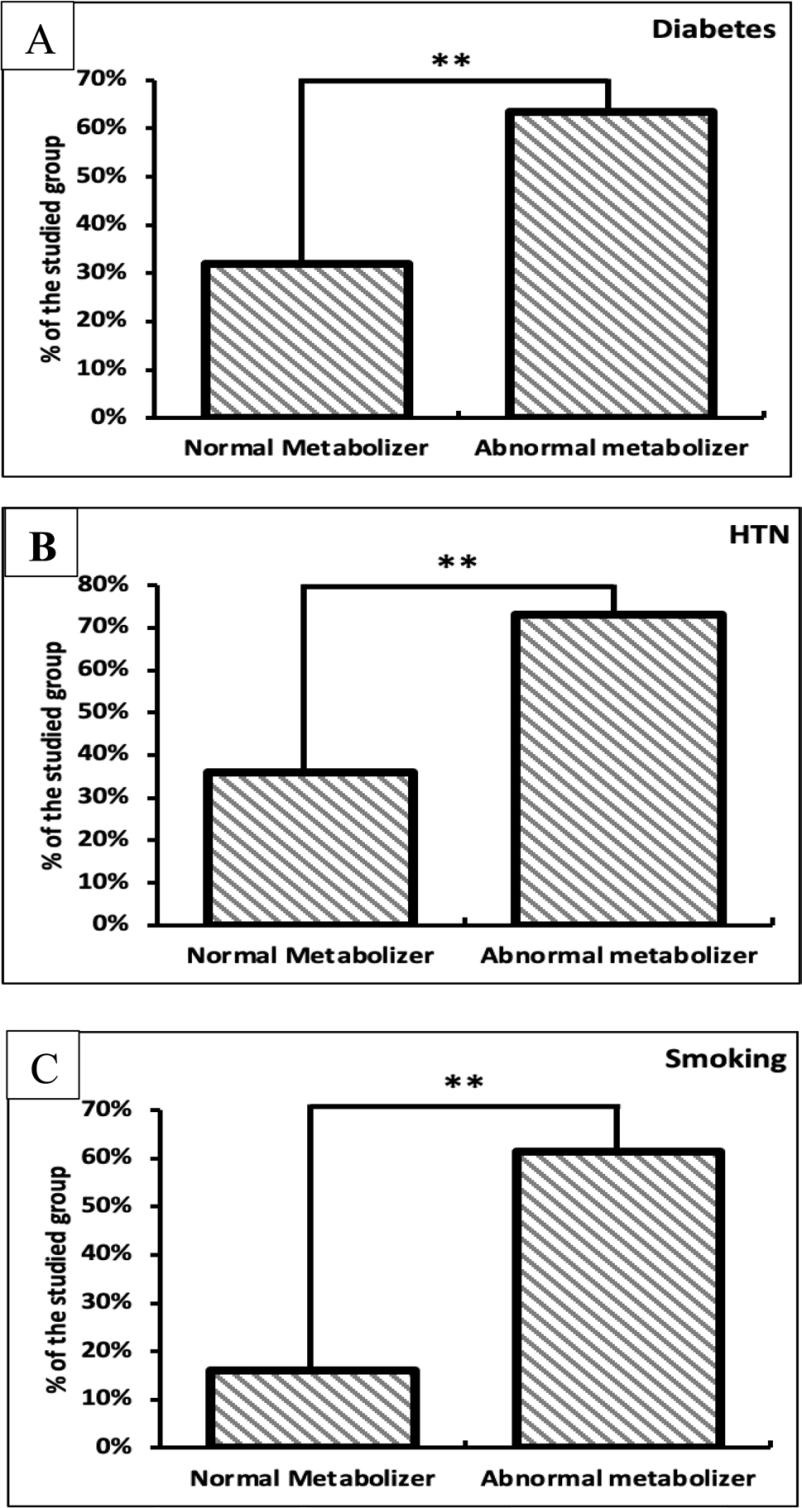
Comparison of normal and abnormal metabolizers according to risk factors

Furthermore, based on the genotyping data, patients were categorized according to their risk factors. Diabetes mellites type 2 significantly affected the patient’s capability to normally metabolize clopidogrel, where out of the 102 patients enrolled in this study, 16 patients (32%) were normal metabolizers, while 33 patients (63.5%) were abnormal metabolizers (*p* = 0.001) (Fig. 3A). Meanwhile, hypertension was also found to affect the metabolizing activity of clopidogrel in patients, where 18 patients (36%) of the study group (n = 102) were normal metabolizers, while 38 (73.1%) were abnormal metabolizers (*p* = 0.001) (Fig. 3B). Smoking also affected the metabolism of clopidogrel in the patients enrolled in this study, where 8 patients (16%) were normally metabolizing clopidogrel, while 32 patients (61.5%) were abnormally metabolizing the drug (*p* = 0.001) (Fig. 3C).

To deeply investigate the effect of normal *versus* abnormal metabolism of clopidogrel in patients, further analysis of follow-up for MACE revealed variations between normal and abnormal metabolizers regarding cardiac events. Data showed a relationship between clopidogrel metabolism and major cardiovascular events. During the follow-up period, one patient with non-ST segmental elevation myocardial infarction was a normal metabolizer to clopidogrel, while no patients with non-STEMI were recorded as abnormal metabolizers, with no significant differences (*p* = 0.984). In addition, one patient with ischemic dilated cardiomyopathy (IDC) was recorded in the normal metabolizer group, while no patients with IDC were recorded as abnormal metabolizers with no significant differences (*p* = 0.984). Furthermore, one patient had MI during the follow-up period from the normal metabolizer group, while seven patients with the same condition were from the abnormal metabolizer group (*p* = 0.047).

**Fig. 4:**
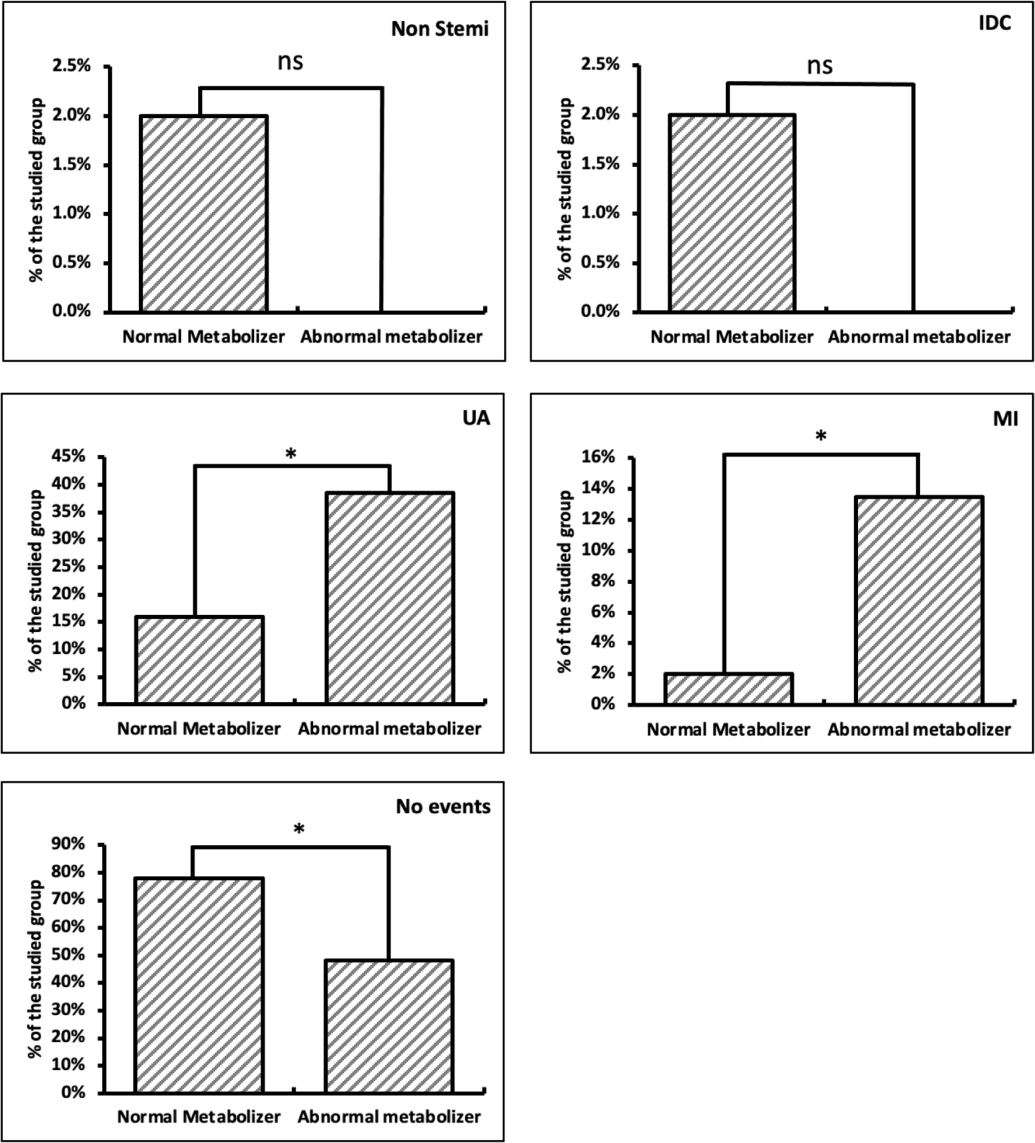
Comparison between normal and abnormal metabolizer according to follow-up for monitoring MACE

Unstable angina was observed in 8 patients from the normal metabolizer group, while 20 patients with the same condition were from the abnormal metabolizer group (*p* = 0.020). No cardiac events were observed in 39 patients belonging to the normal metabolizer group and 25 patients in the abnormal metabolizer group (*p* = 0.004) (Fig. 4).

### Logistic regression

**Fig. 5:**
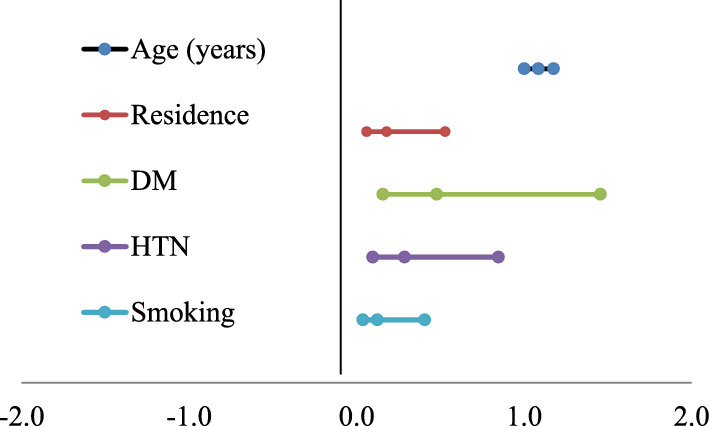
Odds ratio affecting normal metabolizers and abnormal metabolizers

The logistic regression of factors affecting normal metabolizers and abnormal metabolizers was observed. These factors were age, residence, hypertension, and smoking. Other parameters such as the level of blood glucose had no statistically significant effects on the metabolism of clopidogrel. Table 4 and Fig. 5 represent the logistic regression of all parameters affecting the metabolism of clopidogrel.

**Table 4:** Logistic regression of factors affecting normal metabolizer and abnormal metabolizer

Group	B	S.E	Sig	OR	95% C. I	
					Lower	Upper
Age (years)	0.082	0.041	0.045*	1.086	1.002	1.177
Residence	− 1.722	0.553	0.002*	0.179	0.060	0.529
DM	− 0.736	0.567	0.195	0.479	0.158	1.457
HTN	− 1.249	0.553	0.024*	0.287	0.097	0.848
Smoking	− 2.085	0.605	< 0.001**	0.124	0.038	0.407

Finally, for the sake of elucidating the differences in the allelic distribution between the study group and different groups, Table 5 highlights these data.

**Table 5:** Comparison between the study groups and different populations

Polymorphism	Study population allelic frequency	Reported allelic frequency in other populations
CYP2C19*2	15.3%	Iraqi population (15.2%), Saudi Arabian population (15%), Jordanian population (16%), Lebanese population (13.4%), Tunisian population (11.5%), Belgian population (9.1%), Ethiopian population (14%), Japanese population (27.4%), Korean population (20.9%)
CYP2C19*17	N/A	Saudi Arabian population (25.7%), Iraqi population (19.4%)

## Discussion

Cytochromes are a redox-active family of proteins involved in several metabolic activities, including electron transport chain and redox catalysis [23, 24]. *CYP2C19*, a member of this family, plays a crucial role in the metabolism of nearly 18% of the prescribed drugs. Clopidogrel is one of the drugs whose clinical outcome mainly depends on the polymorphism of *CYP2C19* [25, 26]. As commonly prescribed for antiplatelet medication, clopidogrel is associated with MACE risk in patients with loss-of-function *CYP2C19* allele. Till the moment, there are no large randomized controlled trials to evaluate the effectiveness of *CYP2C19* genotyping in patients taking clopidogrel after PCI. However, small-scale clinical studies in different populations might contribute to the growing evidence to support the utility of this test [27].

In the present study, 102 CVD patients were enrolled who had undergone PCI. Patients were males (62.75%) and females (37.255) aged above 60 yr (44.12%) and less than 60 yr (55.88%), and they were living in rural areas (45.1%) or urban areas (45.9%). Those patients were monitored for 1 year. The data obtained showed that 62.75% of the patients underwent no major cardiac events, while 27.45%, 7.87%, 0.98%, and 0.98% underwent unstable angina and myocadiac infarction none-STEMI, and ischemic dilated cardiomyopathy, respectively. After PCI, the 102 Egyptian patients taking clopidogrel were categorized upon genotyping into abnormal metabolizer (50.98%) and normal metabolizer (49.02%). These two categories were subdivided into *1/*2 (15.3%), *1/*3 (1.2%), *1/*17 (35.7%), and *2/*17 (1.2%). These allelic variations in the *CYP2C19* gene were associated with resistance to the clopidogrel antiplatelet activity [28]. But the CYP2C19*17/*17 genotype has been found to increase the clinical response to clopidogrel treatment, and that this genotype is associated with a higher risk of bleeding. Thus, either a loss-of-function allele or a gain-of-function allele might affect the clinical outcomes of patients taking clopidogrel [29], where this is common in different populations due to inter-ethnic variation [21]. For example, in a Lithuanian population (*n* = 54), 33.33% of the patients were NMs (*1/*1), 25.93% were IMs (*1/*2; *2/*17), 27.78% were RMs (*1/*17), 7.4% were UMs (*17/*17), and 5.56% were PMs (*2/*2) [30]. Normal metabolizers account for 33.33%, while abnormal metabolizers account for 66.77%. In another population (Kinh, Vietnam), 100 patients were enrolled in a study to characterize the genotypic variation in the *CYP2C19* gene. Data showed that the allelic distributions were 1%, 2.5%, 20.5%, and 76% for *17, *3, *2, and *1, respectively [31]. This indicates that most of the studied group were normal metabolizers (76%) while 24% were abnormal metabolizers. Although almost equal population size, our study showed fewer normal metabolizers than the Vietnamese population, indicating inter-ethnic variations [30, 32].

The most common allelic variation in *CYP2C19* is *2 and *3 alleles, and it is considered a loss-of-function allele. Patients carrying these alleles are more resistant to clopidogrel, and they are considered abnormal metabolizers for the drug [16, 33, 34]. In our study group, *2 and *3 alleles were 15.3% and 1.2%, respectively (*n* = 102). This might explain the number of patients undergoing MACE during the follow-up period.

In the Egyptian population under study, the CYP2C19*3 allele was represented with a low percentage (1.2%), indicating its insignificant role in regulating the metabolism of clopidogrel, and this allele was not associated with MACE in the Chinese population [35]. However, in a recent study, 190 patients taking clopidogrel were followed up for MACE and genotyped for *CYP2C19* allelic variations, and the results showed that the carriers of loss-of-function *CYP2C19* allele were at increased risk to undergo MACE compared to non-carriers [36].

In the present study, the frequency of CYP2C19*2 was 15.3%, and this was higher than the frequency reported in the Iraqi population (15.2%) [37], Saudi Arabian population (15%) [38], Jordanian population (16%) [39], Lebanese population (13.4%) [40], Tunisian population (11.5%) [41], Belgian population (9.1%) [42], Ethiopian population (14%) [43]. Meanwhile, our reported frequency of CYP2C19*2 was lower than reported in the Japanese population (27.4%) [44], and Korean population (20.9%) [45].

Furthermore, the allelic frequency of CYP2C19*17 reported in our study was 30.39%). The allelic frequency of CYP2C19*17 reported in this study was higher than reported in Saudi Arabian (25.7**%)** [38] and Iraqi populations (19.4%) [37].

The present study showed a significant relationship between age and abnormal metabolism of clopidogrel (*p* = 0.001), while no significant relationship was reported between sex and abnormal metabolism of clopidogrel (*p* = 0.797). Other studies indicated the same profile of clopidogrel metabolism in the elderly [46–48].

Furthermore, in the present study, a significant relationship was reported between residence and abnormal metabolism of clopidogrel (*p* = 0.001), and between diabetes and abnormal metabolism of clopidogrel (*p* = 0.001). These results were consistent with several reports that indicated the significant relationship between diabetes and abnormal metabolism of clopidogrel [49–52].

Smoking is associated with abnormal clopidogrel metabolism [53, 54]. In our study, we reported a significant relationship between cigarette smoking and the abnormal metabolism of clopidogrel. Moreover, we reported that hypertension was associated with the abnormality of clopidogrel metabolism, and this was inconsistent with previously published reports [55–57].

Our data sustain the inter-ethnic variation in clopidogrel metabolism status regarding the phenotype/genotype variation in the *CYP2C19* gene.

## Conclusion

As a prodrug, clopidogrel is converted in the liver to its active form with the aid of hepatic cytochromes. Allelic variation in one of the cytochrome members; *CYP2C19* may cause variabilities in the metabolism of clopidogrel. The present study sheds some light on the inter-ethnic variation in response to the antiplatelet drugs, and this—along with other studies in the same field—might help pave the way to new personalized medicine-based antiplatelets for patients with CVDs.

## Data Availability

All data generated in this work are mentioned in the manuscript.

## Abbreviations

ACS: Acute coronary syndromes
CAD: Coronary artery disease
CBC: Complete blood count
CK-MB: Creatine kinase-MB
CVDs: Cardiovascular diseases
CYP2C19: Cytochrome P450 2C19
CYP450: Cytochromes P450
ECG: Electrocardiogram
HF: Heart failure
IDC: Ischemic dilated cardiomyopathy
IMs: Intermediate metabolizers
IRB: Institutional review board
MACE: Major adverse cardiovascular events
MI: Myocardial infarction
NMs: Normal metabolizers
non-STEMI: Non-ST-segment elevation myocardial infarction
PAD: Peripheral arterial disease
PCI: Percutaneous coronary intervention
PMs: Poor metabolizers
RMs: Rapid metabolizers
SNP: Single-nucleotide polymorphism
STEMI: ST-segment elevation myocardial infarction
TIMI: Thrombolysis in myocardial infarction
UA: Unstable angina
UMs: Ultra-rapid metabolizers
